# Evaluation of the Thermal, Chemical, Mechanical, and Microbial Stability of New Nanohybrids Based on Carboxymethyl-Scleroglucan and Silica Nanoparticles for EOR Applications

**DOI:** 10.3390/nano14080676

**Published:** 2024-04-13

**Authors:** Rubén H. Castro, Laura M. Corredor, Sebastián Llanos, Zully P. Rodríguez, Isidro Burgos, Jhorman A. Niño, Eduardo A. Idrobo, Arnold R. Romero Bohórquez, Karol Zapata Acosta, Camilo A. Franco, Farid B. Cortés

**Affiliations:** 1 Grupo de Investigación en Fenómenos de Superficie—Michael Polanyi, Facultad de Minas, Universidad Nacional de Colombia—Sede Medellín, Medellín 050034, Colombia; kzapata@unal.edu.co (K.Z.A.); caafrancoar@unal.edu.co (C.A.F.); fbcortes@unal.edu.co (F.B.C.); 2 Centro de Innovación y Tecnología—ICP, Ecopetrol S.A., Piedecuesta 681011, Colombia; laura.corredor@ecopetrol.com.co (L.M.C.); jhorman.nino@ecopetrol.com.co (J.A.N.); aidrobo@yahoo.com (E.A.I.); 3 Grupo de Investigación en Química Estructural (GIQUE), Escuela de Química, Universidad Industrial de Santander, Bucaramanga 680002, Colombia; sllanosg@unal.edu.co (S.L.); isidro.burgos@correo.uis.edu.co (I.B.); arafrom@uis.edu.co (A.R.R.B.); 4 PSL Proanálisis, Piedecuesta 680005, Colombia; zuly.rodriguez@pslproanalisis.com

**Keywords:** scleroglucan, carboxymethyl-scleroglucan, nanohybrid, biodegradation, EOR

## Abstract

Scleroglucan (SG) is resistant to harsh reservoir conditions such as high temperature, high shear stresses, and the presence of chemical substances. However, it is susceptible to biological degradation because bacteria use SG as a source of energy and carbon. All degradation effects lead to viscosity loss of the SG solutions, affecting their performance as an enhanced oil recovery (EOR) polymer. Recent studies have shown that nanoparticles (NPs) can mitigate these degradative effects. For this reason, the EOR performance of two new nanohybrids (NH-A and NH-B) based on carboxymethyl-scleroglucan and amino-functionalized silica nanoparticles was studied. The susceptibility of these products to chemical, mechanical, and thermal degradation was evaluated following standard procedures (API RP 63), and the microbial degradation was assessed under reservoir-relevant conditions (1311 ppm and 100 °C) using a bottle test system. The results showed that the chemical reactions for the nanohybrids obtained modified the SG triple helix configuration, impacting its viscosifying power. However, the nanohybrid solutions retained their viscosity during thermal, mechanical, and chemical degradation experiments due to the formation of a tridimensional network between the nanoparticles (NPs) and the SG. Also, NH-A and NH-B solutions exhibited bacterial control because of steric hindrances caused by nanoparticle modifications to SG. This prevents extracellular glucanases from recognizing the site of catalysis, limiting free glucose availability and generating cell death due to substrate depletion. This study provides insights into the performance of these nanohybrids and promotes their application in reservoirs with harsh conditions.

## 1. Introduction

The ever-increasing demand for energy coupled with the depletion of easily accessible oil reserves has driven the oil and gas industry to explore innovative technologies to enhance oil recovery from existing reservoirs. In this pursuit, researchers have investigated advanced materials that exhibit unique properties that improve the efficiency of enhanced oil recovery (EOR) processes. Integrating biopolymers like scleroglucan and nanotechnological innovations promises to play a pivotal role in maximizing oil recovery and ensuring energy security while minimizing environmental impact.

Scleroglucan is an extracellular polysaccharide produced by fungi (*Sclerotium glucanicum*). As a high-molecular-weight biopolymer, scleroglucan has demonstrated excellent stability under harsh reservoir conditions. Its non-toxic and biodegradable nature enhances its appeal for sustainable and environmentally friendly oil recovery processes. Scleroglucan is a neutral, in aqueous phase that presents a conformation with a triple helix, semi-rigid, rod-like structure, without the formation of aggregates due to *D*-glycosidic side groups. Due to its non-ionic nature, the viscosity of the solutions is stable at high ionic forces and different pH values [1,2,3,4]. Studies of the scleroglucan stability demonstrated that its solutions retained more than 95% of the original viscosity in several months at temperatures up to 115 °C. Also, scleroglucan is unimpacted by common reservoir salts at concentrations up to 30,000 ppm (divalent and trivalent cations) [2,5,6].

Kozlowicz et al. [2] evaluated the performance of EOR grade scleroglucan solutions under laboratory (up to 115 °C and 180 days, 175,000 s^−1^, 500 ppm Fe^2+^, 200 ppm H_2_S) and reservoir conditions (Ø = 19.7%, K= 805 mD, 42°API oil, 8000 ppm TDS, 35 ppm TSS and 82 °C) for the Adena field located in the United States. The evaluation of the injection in the field demonstrated that scleroglucan was thermally, chemically, and mechanically stable under the evaluated conditions. However, the monitoring of bacterial activity in this field test was not reported. Kozlowicz et al. [7] demonstrated that despite the lability of scleroglucan against microbiological degradation under reservoir conditions, biocides like glutaraldehyde, formaldehyde, tetrakis(hydroxymethyl)-phosphonium sulfate (THPS), and 1,3,4,6-tetrakis(hydroxymethyl)tetrahydroimidazo-[4,5-d]imidazole-2,5(1H,3H)-dione (TMAD) could be used without impacting the scleroglucan solution viscosity. Anaerobic cultures isolated from hydrocarbon-related environments were incubated with SG prepared in brine at >70,000 ppm; the results showed an SG viscosity reduction of 60% and 1% with an increase in glucose consumption due to the action of microorganisms of 80% and 0% when the systems were incubated at 30 and 60 °C, respectively. The results show that the high temperature, high salinity, and harshest anoxic conditions represent a more suitable environment to guarantee the microbiological stability of SG viscosity. However, the conditions could favor microbial growth in the zone near the wellbore (which is colder) and in the reservoirs with low salinity. It has become the most important target for antimicrobial controls, so nanotechnology has become a promising option for SG self-preservation and reinforcing viscous networks.

Nanoparticles offer unique characteristics that can address the limitations of EOR polymers or biopolymers [8,9]. Nanoparticles can be incorporated into polymers by dispersing them into polymer solutions (through electrostatic binding, hydrogen bonding, or hydrophobic forces) [10,11,12,13] or by grafting the polymeric chains onto the nanoparticle’s surface [14,15,16]. Understanding the effect of nanoparticles on the resistance of scleroglucan to harsh conditions is crucial for tailoring EOR solutions. For this reason, in this study, the performance of two new nanohybrids under different salinities (0–40,000 ppm NaCl concentration), temperatures (30–100 °C), pH (2–13), and shear rates (0–320,000 s^−1^) was evaluated. Also, the susceptibility of scleroglucan to microbial degradation was measured at 60 °C and 1311 ppm (TDS). The results showed that incorporating the nanoparticles into the SG structure did not affect the biopolymer’s thermal, chemical, and mechanical resistance but reduced its microbial sensitivity. This research refines the operational window where the nanohybrids maintain their viscosifying power and proves beneficial for EOR applications.

## 2. Materials and Methods

### 2.1. Materials

**Figure 1:**
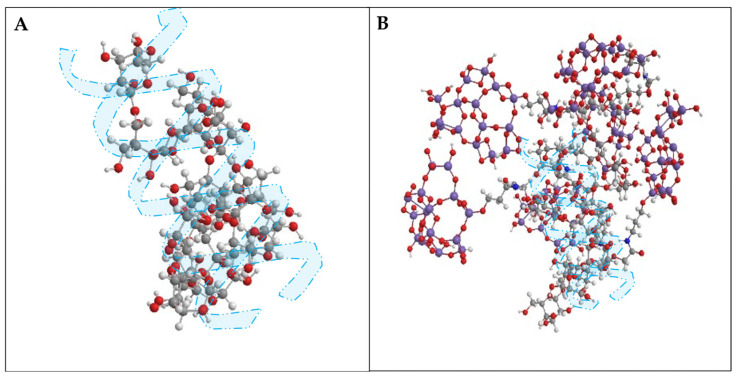
Proposed tridimensional conformation of (**A**) SG and (**B**) NH-B. Oxygen atoms are represented in red, carbon in gray, hydrogen in white, nitrogen in blue, and silicon in purple.

The biopolymer used in this study was commercial EOR-grade scleroglucan with 99% purity and MW~4.5 × 10^6^ Da. The nanohybrids, NH-A and NH-B, were obtained by modifying SG of two degrees of carboxymethyl substitution, DS = 0.22, and DS = 0.44, with amino-functionalized SiO_2_ nanoparticles (SiO_2_ APTES, 99+%, 20 nm, 120 m^2^/g, amphiphilic, manufactured by Nanostructured & Amorphous Materials, Inc., Los Alamos, NM, USA. To synthesize the nanohybrids, the amidation reaction of carboxymethyl-scleroglucans [17] and commercial amino-functionalized silica nanoparticles was carried out through carbodiimide-assisted coupling, as reported in our previous work [10]. Figure 1 presents the tridimensional conformation of SG (Figure 1A) and NH-B (Figure 1B) obtained in Chem3D Software (version 18.1.0.535). The structure of the SG repetitive unit and degree of branching are similar to those reported in the literature [18].

Sterile injection brine, synthetic brine, or injection brine (conductivity = 2480 μS/cm, TDS = 1311 ppm, and pH = 8.17), and 100 ppm of a commercial glutaraldehyde-based biocide was used for anaerobic biodegradation tests. The synthetic brine was prepared with 0.829 g/L sodium chloride (NaCl, 99.5% pure, Merck Millipore, Sigma-Aldrich, St. Louis, MO, USA), 0.035 g/L potassium chloride (KCl, 99.5% pure, Merck Millipore, Sigma-Aldrich, MO, USA), 0.069 g/L magnesium chloride (MgCl_2_·6H_2_O, 99% pure, Merck Millipore, Sigma-Aldrich, St. Louis, MO, USA), and 0.331 g/L calcium chloride (CaCl_2_·2H_2_O, 99% pure, Merck Millipore, Sigma-Aldrich, St. Louis, MO, USA), and type II water (pH ≈ 7).

### 2.2. Methods

#### 2.2.1. Biopolymer Solutions Preparation

All solutions were prepared as described by Abraham and Sumner [19], and Castro et al. [4]. First, the brine was placed on a magnetic stirrer with the vortex adjusted to 90% of the liquid height (500 rpm); then, each powder was slowly sprinkled into the shoulder of the vortex (over a period of 10 to 15 min to avoid the formation of agglomerates). Finally, the solutions were stirred at 800 rpm, heated at 40 °C for 10 min, and homogenized for 5 min using a high-performance immersion blender at 20,000 rpm (T 25 digital ULTRA-TURRAX®, IKA, Sao Paulo, Brazil). The stock solutions were diluted with brine to achieve the target concentration (1000 ppm for SG, 1200 ppm for NH-A, and 1400 ppm for NH-B).

For the anaerobic biodegradation tests, the solutions were prepared with sterile injection brine, synthetic brine, or injection brine filtered through a 0.45 μm MCE membrane filter (Merck Millipore, Sigma-Aldrich, St. Louis, MO, USA). The biopolymer solutions were prepared at 930 ppm for SG, 1200 mg/L for NH-A, and 1400 ppm for NH-B.

#### 2.2.2. Viscosity Measurements

A DV2TTM viscosimeter (AMETEK Brookfield, MA, USA) with ULA-type geometries (µ < 100 cP, Accuracy: ±1.0% of range, Repeatability: ±0.2%) was used to determine the nominal viscosities of all samples at 30, 45, and 60 °C and a shear rate of 6 RPM (equivalent to 7.3 s^−1^ for the ULA geometry).

#### 2.2.3. Rheological Behavior

The flow curves of all solutions were measured using an Anton Paar MCR502 rheometer (Anton Paar GmbH, Gratz, AUT) with a Peltier system, pressure cell, and coaxial cylinder geometry. The rheology data matched the Carreau–Yasuda model [20,21,22]:

(1) $$\mu =\eta_{\infty }+(\eta_{0}-\eta_{\infty }){\left[1+(\lambda \dot{\gamma }{)}^{\alpha }\right]}^{(n-1)/\alpha }$$

The Carreau–Yasuda model describes the behavior of non-Newtonian fluids as a function of the zero and the infinite shear viscosity (cP), $\eta_{0}$ and $\eta_{\infty }$, the shear rate, $\dot{\gamma }$ (1/s), the power law exponent n (dimensionless), the relaxation parameter λ (s), and the transition parameter α (dimensionless) [20].

#### 2.2.4. Filter Ratio (FR)

All samples were filtered according to the recommendations in API RP 63 [23]. For this test, 300 mL of each solution were filtered through a 1.2 and 5 µm Millipore cellulose (Merck Millipore, Sigma-Aldrich, St. Louis, MO, USA) filter at a constant pressure of 30 psi. The collection time (t) the filtered fluid was recorded at 300, 200, and 100 mL, and the FR was calculated as follows:

(2) $$FR=\frac{t_{300}-t_{200}}{t_{200}-t_{100}}$$

#### 2.2.5. Thermal Stability

The stability of the NH-A and NH-B solutions was evaluated at different temperatures (30 °C to 100 °C) and 531 psi over the range 4–424 s^−1^, as described in the rheological behavior Section 2.2.3.

#### 2.2.6. Mechanical Stability

The mechanical stability is determined as the viscosity retention ratio, and it is calculated by dividing the apparent viscosity of the polymer or nanohybrid solution after and before shearing (at 7.3 s^−1^ at 30 °C) [23]. The shearing process was performed by injecting the samples through a shearing device with the principle of flow through small nozzles (ID 1/8″ and 1/16″). Shearing was conducted by applying 30 to 800 psi pressure (nitrogen). The shear rates ($\dot{\gamma }$) applied to the solutions (between 0 and 320,000 s^−1^) were calculated with the internal radius of the capillary (R) and flow rates (Q) according to the following equation:

(3) $$\dot{\gamma }=\frac{4Q}{\pi R^{3}}$$

#### 2.2.7. Chemical Stability: Effect of pH and Electrolytes

The effect of the pH (2–13) and NaCl concentration (0–40,000 ppm) on the viscosity of the SG and NH solutions was also evaluated. The viscosity measurements were carried out in a viscosimeter at 7.3 s^−1^ and 30 °C.

#### 2.2.8. Microbial Stability: Anaerobic Biodegradation Tests

Table 1 describes the samples used to determine the biological degradation of biopolymers, SG, and nanohybrids (NH) solutions under anaerobic conditions.

Rodriguez et al. [24] reported the methodology used for the anaerobic biodegradation tests. All biopolymer solutions (sterile water, synthetic brine, injection water with and without biocide) and injection water were bubbled with sterile nitrogen to assimilate the anaerobic environment until the dissolved oxygen concentration was close to 0 mg/L. Then, 100 mL of each biopolymer solution was stored in 150 mL glass bottles with plastic caps fitted with butyl rubber septum. Biopolymer solution samples were bubbled again with sterile nitrogen to ensure an oxygen-free system. Finally, viscosity and bacteria count were measured in established periods.

The total anaerobic bacteria count was obtained with the serial dilution technique by extinction of anaerobic bacteria (thioglycollate broth test) recommended by NACE TMO 194 [25]. The RW’s initial microbial load was 1.0 × 10^3^ total anaerobic bacteria/mL. The incubation process of biotic solutions (exposed to the RW’s initial microbial load diluted at different concentrations) was carried out at 60 °C and 100 rpm in glass bottles. All samples were tested in duplicate. The concentration of bacteria was determined using microbiological techniques, such as dilution by extinction of total anaerobic bacteria due to using of a biocide solution in some matrices [25]. Other methodologies, e.g., molecular tools such as real-time PCR, do not allow a distinction between viable and non-viable cells due to the action of the biocide.

## 3. Results

### 3.1. Rheological Behavior

**Figure 2:**
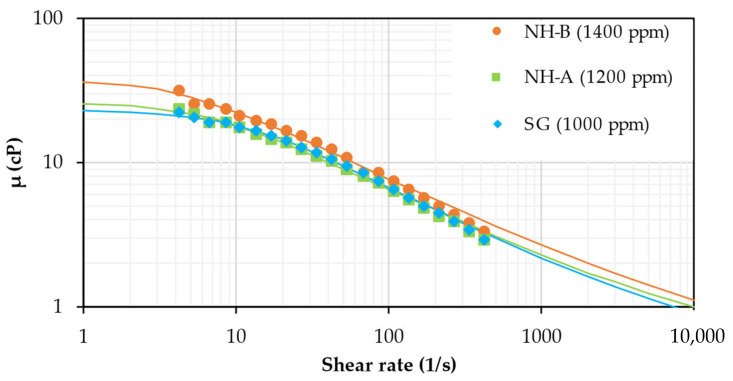
Flow curves of 1200 ppm NH-A, 1400 ppm NH-B, and 1000 ppm SG solutions in brine (TDS = 1311 ppm) at 531 psi and 100 °C.

Figure 2 shows the non-Newtonian behavior of the SG and both nanohybrids. The concentrations of the nanohybrids and SG solutions were selected to reach mobility ratios close to one and minimize the viscous fingering in future coreflooding tests. The mobility ratio (M) were calculated from Equation (4):

(4) M=KrwμwKroμo

where $K_{rw}$ is the water-effective permeability, $\mu_{w}$ is the water viscosity, $K_{ro}$ is the oil-effective permeability, and $\mu_{o}$ is the oil viscosity.

The solution viscosities (µ) should be close to 18 cP at 7.3 s^−1^ and 100 °C to reach mobility ratio values ~1 [4]. The concentration required to achieve the target viscosity is 1000 ppm for SG, 1200 ppm for NH-A, and 1400 ppm for NH-B. Both nanohybrids exhibited similar rheological behavior to scleroglucan [14], but higher concentration is needed to achieve the target viscosity (20% and 40% more concentration compared to NH-A and NH-B, respectively). The viscosity reduction is attributed to the carboxymethylation and amidation reactions, where the hydroxyl groups on the SG structure (contributing to SG viscosity) are substituted by carboxyl groups and amino-functionalized silica nanoparticles.

The Carreau–Yasuda model parameters of SG, NH-A, and NH-B are shown in Table 2. The zero shear viscosity increased, and the shear thinning behavior (n increased) of the SG solutions was reduced by increasing the nanoparticle content in the nanohybrid (6.07 ppm NH-B > 5.7 ppm NH-A) because the formation of an NP-SG three-dimensional network hinders the alignment of the SG chains at an applied shear rate. Also, these parameters are affected by the difference in the NH-A and NH-B solutions concentration (NH-B > NH-A).

The relaxation parameter and the infinite shear viscosity were fixed at 1.5 s and 0.282 cP (water viscosity at 100 °C), respectively, and the structure factor associated with biopolymers [26].

### 3.2. Filter Ratio (FR)

**Figure 3:**
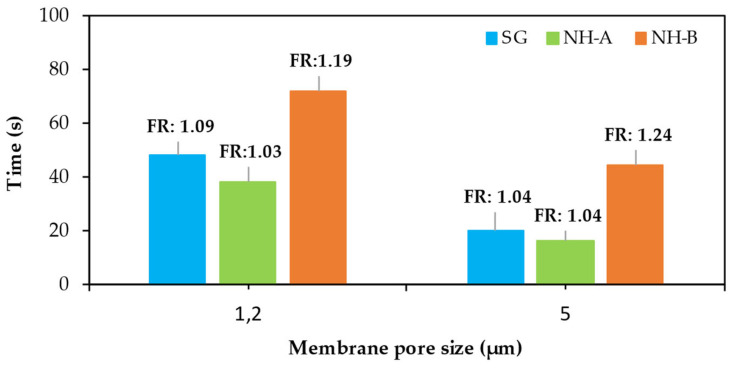
Filter ratio of 1200 ppm NH-A, 1400 ppm NH-B, and 1000 ppm SG solutions in brine (TDS = 1311 ppm) through a 1.2 and 5 µm Millipore cellulose filter at 30 psi and 30 °C.

Most of the filter ratios of the NH-A, NH-B, and SG solutions were below 1.2 (Figure 3) [27], which means that the samples exhibited good filterability and no plugging tendency. This is an advantage compared to synthetic polymers with high molecular weights at lower concentrations. The NH-B solutions exhibited the highest filter ratios due to their higher nanoparticle content, which changed their hydrodynamic configuration compared to the NH-A. Good or bad filterability via the classical filter ratio test is not a token of good or bad propagation in porous media, and it is required to perform core flooding tests [26]. These tests will be included in our future experimental work.

### 3.3. Mechanical Stability

**Figure 4:**
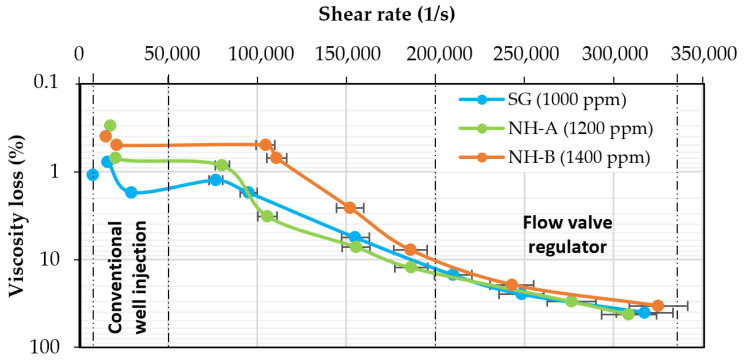
Viscosity loss as a function of shear rate for 1200 ppm NH-A, 1400 ppm NH-B, and 1000 ppm SG solutions in brine (TDS = 1311 ppm) at 30 °C.

The viscosity loss of both nanohybrids is lower than that of the SG solution below 50,000 s^−1^ (Figure 4). The NP-SG three-dimensional network of the nanohybrids can explain its improved mechanical shearing resistance. This network can absorb and distribute the energy applied to the sample during the shearing process, impacting the viscosity behavior. Between 50,000 and 180,000 s^−1^, only the NH-B exhibited higher mechanical shearing resistance than the SG. As stated, this is attributed to the difference in the silica content in the nanohybrids, which modifies their hydrodynamic configuration. After 180,000 s^−1,^ all samples had viscosity losses greater than 40%. Both nanohybrids showed lower viscosity losses than synthetic polymers reported in the literature, which had values of 80%.

### 3.4. Chemical Stability: Effect of pH and Electrolytes

**Figure 5:**
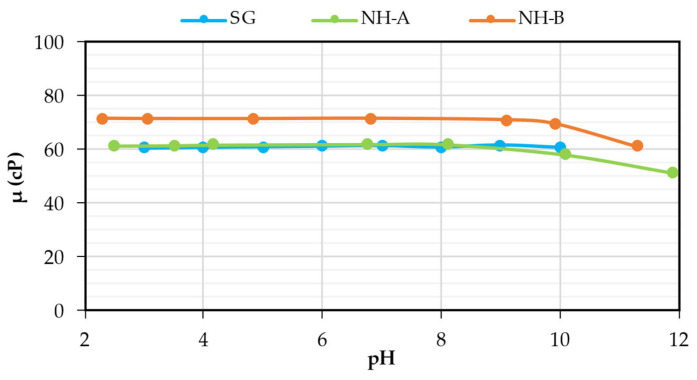
Effect of pH on the viscosity of the 1200 ppm NH-A, 1400 ppm NH-B, and 1000 ppm SG solutions in brine (TDS = 1311 ppm) at 30 °C and 7.3 S^−1^.

According to Figure 5, the viscosity of all solutions is stable within the broad range of pH evaluated (2–12). This means that the pH stability of the SG was not affected by carboxymethylation (CMS products) and amidation reactions (NH products). However, as for SG, the triplex denaturation and the chemical degradation of its chains occur in both nanohybrids at pH > 12. A pronounced decrease in the apparent viscosity and loss of pseudoplastic behavior of the solutions evidence denaturation. Denaturation occurs when the strength of interstrand H-bonds inside the helical core of the SG (with influence from hydrophobic forces) decreases below a critical limit, causing the dissociation of the SG triplex into random coils [28]. At high pH, these forces weaken due to the high charge density, leading to electrostatic repulsions between the strands.

**Figure 6:**
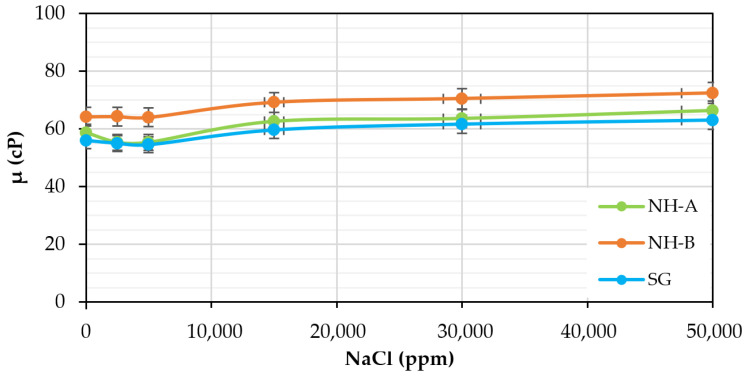
Effect of NaCl concentration on the viscosity of the 1200 ppm NH-A, 1400 ppm NH-B, and 1000 ppm SG solutions in brine (TDS = 1311 ppm) at 30 °C and 7.3 s^−1^.

According to Figure 6, all samples exhibited high salt tolerance, which concurs with previous results reported in the literature for the SG [2,7], due to nanohybrids also exhibiting a non-ionic nature similar to scleroglucan which does not have intrinsic charges in its structure that interact with ions present in the brine. Unlike synthetic polymers, where the viscosity decreases considerably because monovalent and divalent cations neutralize the anionic charges of the HPAM chains, the synthesized products are good candidates as EOR agents for reservoirs with high or low salinities.

### 3.5. Microbial Stability: Anaerobic Biodegradation Tests

**Figure 7:**
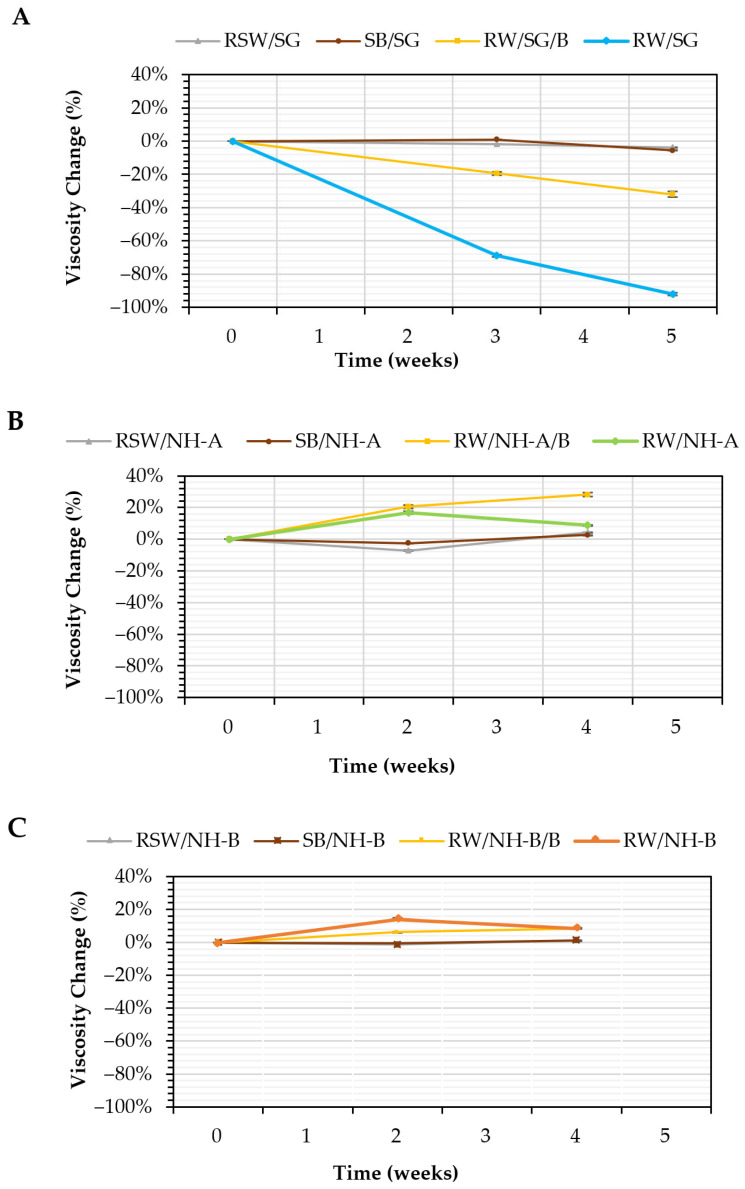
Viscosity change as a function of time of (**A**) SG solutions in sterilized injection water (RSW/SG), synthetic brine (SB/SG), injection water with biocide (RW/SG/B) and without biocide (RW/SG) [24], (**B**) NH-A solutions in sterilized injection water (RSW/NH-A), synthetic brine (SB/NH-A), injection water with biocide (RW/NH-A /B) and without biocide (RW/NH-A), and (**C**) NH-B solutions in sterilized injection water (RSW/NH-B), synthetic brine (SB/NH-B), injection water with a biocide (RW/NH-B/B) and without biocide (RW/NH-B). All systems at TDS = 1311 ppm, 7.3 s^−1^, and 30 °C.

In our previous work [24], the viscosity change as a function of time of the SG solutions in sterilized injection water (RSW/SG), synthetic brine (SB/SG), and injection water with biocide (RW/SG/B) and without biocide (RW/SG) was reported as shown in Figure 7A. Likewise, Figure 7B,C show the viscosity changes as a function of time for NH-A and NH-B nanohybrids.

The SG solutions in sterile water (RSW/SG) and synthetic brine (SB/SG) showed no significant differences in the viscosity changes after five weeks (*p*-value = 0.345 and 0.242, respectively). This result was attributed to the absence of microorganisms (-abiotic systems- synthetic and sterile media) and SG’s non-ionic nature, making them salt-resistant [29]. In contrast, the viscosity reduction of SG solution in injection water without biocide (RW/SG) and with biocide (RW/SG/B) was 92% (from 49.5 to 3.9 cP) and 32% (from 49 to 33.3 cP), respectively, suggesting growth of endogenous bacteria and use of SG for their metabolism [24]. In contrast, the viscosity of the NH-A and NH-B in injection water without the biocide showed an increase in viscosity of 9%, changing from 69 to 75 cP for RW/NH-A and from 74.8 to 81.2 cP for RW/NH-B. In the presence of biocide, the viscosity of RW/NH-A/B and RW/NH-B/B increased by 28% (from 68.9 to 88.3 cP) and 9% (from 74.9 to 81.3 cP), respectively. The changes in each system are shown in Table 3.

The study was also reported that the anaerobic bacterial count in the abiotic controls with sterile water (RSW/ SG) and synthetic brine (SB/SG) was <1.0 × 10^1^ bacteria/mL during the time of evaluation (Table 4). The initial bacterial count of the RW/SG/B solution was <1.0 × 10^1^ bacteria/mL but increased to 1.0 × 10^1^ bacteria/mL in week 3, indicating that the biocide was not 100% effective. On the other hand, the anaerobic bacterial count in the RW/SG solution increased from 1.0 × 10^2^ to 1.0 × 10^3^ bacteria/mL in week 5. Finally, the initial bacterial count of RW brine was 1.0 × 10^2^ bacteria/mL and decreased to <1.0 × 10^1^ bacteria/mL in week five due to the lack of carbon sources that enable bacterial growth [30].

The results obtained for RW/NH-A and RW/NH-B (<1.0 × 10^1^ bacteria/mL at week 4) revealed the antimicrobial power of nanohybrids, which correlates with an increase in the viscosifying power with aging (9% for both nanohybrids). Finally, the systems with biocides showed antimicrobial synergy in the presence of the nanohybrids, RW/NH-A/B and RW/NH-B/B, with a negligible bacterial growth <1.0 × 10^1^ bacteria/mL in week 4, but in their absence, for example, for RW/SG/B, the bacterial count increased to 1.0 × 10^1^ bacteria/mL in week 3, indicating that the biocide were not 100% effective. The results also correlate with the viscosity changes in these systems, in which a loss of viscosity for RW/SG/B of 32% was evident, but a reinforcement of the viscous network in the presence of the RW/NH-A/B and RW/NH-B/B nanohybrids, with viscosity increases of 28% and 9%, respectively.

**Figure 8:**
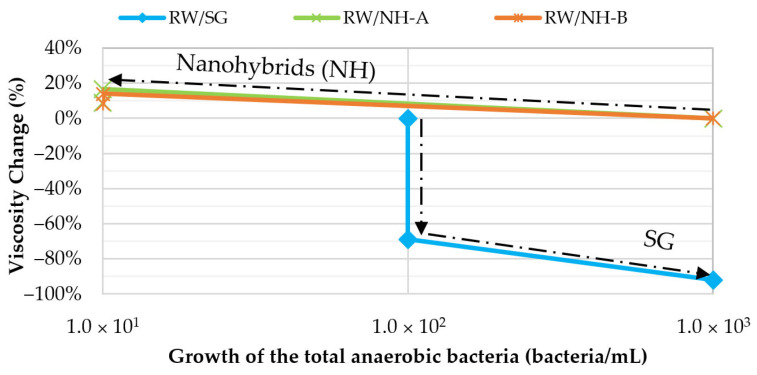
Viscosity loss as a function of the growth of the total anaerobic bacteria (incubated at 60 °C) in a biotic system (injection water) without biocide for RW/SG, RW/NH-A, and RW/NH-B at TDS = 1311 ppm, 7.3 s^−1^ and 30 °C.

Figure 8 presents the viscosity loss as a function of the total growth of the anaerobic bacteria for all biotic systems analyzed to understand the relationship between bacterial count and the viscous properties of SG-based solutions.

SG showed a viscosity loss change from 69% to 92% with an increase in bacterial count from 1.0 × 10^2^ bacteria/mL to 1.0 × 10^3^ bacteria/mL in 5 weeks; however, for both nanohybrids, the bacteria concentration decreased from 1.0 × 10^3^ bacteria/mL to <1.0 × 10^1^ bacteria/mL after the four weeks, with an increase in the solution viscosities of 9%. It shows that the chemical modifications of the SG influenced the control of biodegradation of the biopolymer. Nanohybrids showed not only the best antimicrobial effect but also a better reinforcement of the viscous network; this could be due to dead bacterial strains in the polymer solutions that function as suspended solids, increasing the viscosity of the systems.

Evidence demonstrates that suspended particles form networks with strong interactions between them or with the continuous phase [31,32,33]. Given the anionic nature of the dead bacterial walls, electrostatic interactions with the electropositive zones of the water molecules and the amino groups of the nano-hybrids are highly probable, which allows the reinforcement of the viscoelastic network [34]. These results explain why, in synergistic systems, biocides plus nanohybrids, where the antimicrobial power was greater (<1.0 × 10^1^ bacteria/mL at week 0), there have been more significant increases in viscosity (up to 28%).

The viscosity loss of non-sterile systems based on SG was attributed to the capacity of bacteria to use SG as a carbon source. Scleroglucan is a source of glucose that will be converted to pyruvate by glycolysis during primary metabolism, promoting bacterial count. To obtain glucose monomers from SG, bacteria first release extracellular glucanases, which break down the β-*D*-(1-3)-glucopyranosyl chain [30,35] and their bonds β-*D*-(1-6)-glucopyranosyl groups, depolymerizing SG and reducing its viscosifying power.

**Figure 9:**
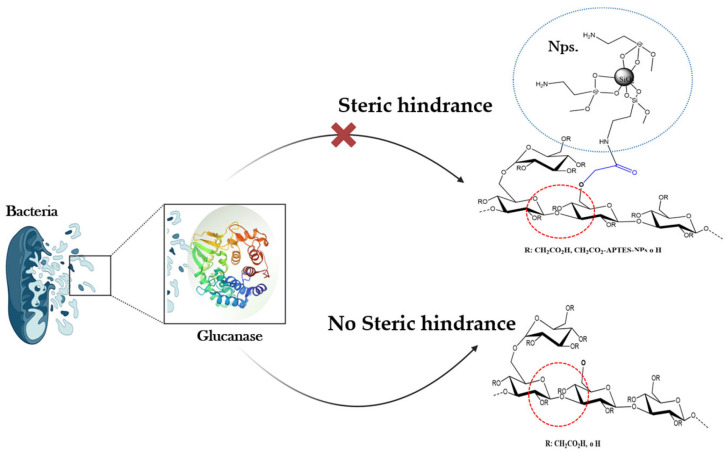
Proposed inhibition mechanism of nanohybrids against bacterial proliferation by starvation.

To understand the antimicrobial role of nanohybrids, bacteria from injection waters were cultured with the SiO_2_ nanoparticles (SiO_2_ APTES NPs, 99+%, 20 nm, 120 m^2^/g, amphiphilic), and cell viability was determined through the phenol red test recommended [25]. The technique is based on the change in color from red to yellow due to the medium’s pH modification by the acid produced by viable bacteria (Table 5). The negative control in the absence of biocide and nanoparticles was used to evaluate the natural growth of the bacteria. After 15 days, no growth inhibition or bacterial death was observed at the evaluated NPs concentrations (25–150 ppm). In contrast, the positive control in the presence of the biocide (100 ppm) without SiO_2_ NPs inhibited bacterial growth. It means that the antimicrobial action associated with NH-A and NH-B products is not related to an antibacterial effect of the NPs but to their ability to interfere with the use of SG as a carbon source by steric hindrance (Figure 9).

During SG anaerobic metabolism, bacteria release glucanases to convert SG to glucose monomers [36]. The metabolic fate of glucose molecules is conversion to pyruvate and other lower molecular weight molecules with the simultaneous release of energy molecules [37]; however, in the presence of nanohybrids, extracellular glucanases are unable to recognize the site of action [38,39,40], decreasing the supply of glucose units in the medium and leading to cell death due to starvation [36,41]. There is evidence that many polymers are chemically modified to increase microbiological resistance [42].

## 4. Conclusions

The SG and new nanohybrids are highly viscosifying products, resistant to chemical (up to 40,000 ppm of NaCl and pH between 2 and 13), thermal (up to 100 °C), and mechanical (up to 320,000 s^−1^) degradation.

The SG microbial degradation test showed that bacteria grew in the biotic control in injection water without biocide (RW/SG), causing viscosity reductions because bacteria use the SG as an energy and carbon source. Additionally, it was found that the biocide did not mitigate the total bacterial growth in the biotic control in injection water RW/SG/B.

NH-A and NH-B microbial degradation test showed bacterial control in the biotic control in injection water with and without biocide (RW/NH-A and RW/NH-B) and increased the solution viscosities. The bacterial control of the nanohybrid solutions was attributed to the steric hindrance given by the modifications with nanoparticles to SG and not to a direct antibacterial action of the nanostructures. The inability of bacteria to hydrolyze SG using glucanase limits the availability of free glucose, generating cell death due to substrate depletion. Likewise, the gradual presence of non-active suspended solids, such as dead cells, increases the viscosity of the aged systems.

In conclusion, covalent grafting of the nanoparticles into the SG structure reduces the biopolymer’s microbial sensitivity while maintaining its thermal, chemical, and mechanical resistance. This indicates that the nanohybrids could be good candidates for EOR processes.

## Figures and Tables

**Table 1:** Solutions and brines (TDS = 1311 ppm) used in the anaerobic degradation tests of the SG, NH-A, and NH-B.

Sample	Abbreviation	Description
Sterile injection water with biopolymer	RSW/bio	Abiotic control in sterile injection water
Synthetic brine with biopolymer	SB/bio	Abiotic system in synthetic brine
Injection of water with biopolymer	RW/bio	Biotic control in injection water without biocide
Injection of water with biopolymer plus biocide	RW/bio/B	Biotic system in injection water with biocide
Injection water	RW	Biotic control without biopolymer

**Table 2:** Rheological parameters of 1200 ppm NH-A, 1400 ppm NH-B, and 1000 ppm SG solutions in brine (TDS = 1311 ppm).

Parameter	SG	NH-A	NH-B
n	0.4360	0.4801	0.4854
$\eta_{0}$	22.9530	26.2618	37.7623
λ	0.0948	0.1619	0.2421
RMSE (%)	2.66	3.59	4.58

**Table 3:** Viscosity change (%) in SG, NH-A, and NH-B solutions in brine after a few weeks (TDS = 1311 ppm).

Sample	Abbreviation	Viscosity Change at 4/5 Weeks (%)
Sterile injection water with biopolymers (abiotic system)	RSW/SG	−4
	RSW/NH-A	+4
	RSW/NH-B	+1
Synthetic brine with biopolymers (abiotic system)	SB/SG	−6
	SB/NH-A	+3
	SB/NH-B	+1
Injection of water with biopolymers (biotic control)	RW/SG	−92
	RW/NH-A	+9
	RW/NH-B	+9
Injection of water with biopolymers plus biocide (biotic system)	RW/SG/B	−32
	RW/NH-A/B	+28
	RW/NH-B/B	+9

**Table 4:** Total growth of the anaerobic bacteria (bacteria/mL) in SG, NH-A, and NH-B solutions in brine (TDS = 1311 ppm) after few weeks.

Sample Type	Abbreviation	Total Growth of the Anaerobic Bacteria (Bacteria/mL)		
		0 Weeks	2–3 Weeks	4–5 Weeks
Sterile injection water with biopolymer (abiotic control)	RSW/SG	<1.0 × 10^1^	<1.0 × 10^1^	<1.0 × 10^1^
	RSW/NH-A	<1.0 × 10^1^	<1.0 × 10^1^	<1.0 × 10^1^
	RSW/NH-B	<1.0 × 10^1^	<1.0 × 10^1^	<1.0 × 10^1^
Synthetic brine with biopolymer (abiotic system)	SB/SG	<1.0 × 10^1^	<1.0 × 10^1^	<1.0 × 10^1^
	SB/NH-A	<1.0 × 10^1^	<1.0 × 10^1^	<1.0 × 10^1^
	SB/NH-B	<1.0 × 10^1^	<1.0 × 10^1^	<1.0 × 10^1^
Injection of water with biopolymer (biotic control)	RW/SG	1.0 × 10^2^	1.0 × 10^2^	1.0 × 10^3^
	RW/NH-A	1.0 × 10^3^	<1.0 × 10^1^	<1.0 × 10^1^
	RW/NH-B	1.0 × 10^3^	<1.0 × 10^1^	<1.0 × 10^1^
Injection of water with biopolymer plus biocide (biotic system)	RW/SG/B	<1.0 × 10^1^	1.0 × 10^1^	1.0 × 10^1^
	RW/NH-A/B	<1.0 × 10^1^	<1.0 × 10^1^	<1.0 × 10^1^
	RW/NH-B/B	<1.0 × 10^1^	<1.0 × 10^1^	<1.0 × 10^1^
Injection water	RW	1.0 × 10^3^	1.0 × 10^2^	1.0 × 10^1^

**Table 5:** Viability of endogenous bacteria from the injection water at 60 °C and different concentrations of amino-functionalized SiO_2_ nanoparticles (25–150 ppm) after 15 days.

Biological System						
Negative Control (Injection Water)	Injection Water (Bacteria) with 25 ppm NPs	Injection Water (Bacteria) with 50 ppm NPs	Injection Water (Bacteria) with 78 ppm NPs	Injection Water (Bacteria) with 100 ppm NPs	Injection Water (Bacteria) with 150 ppm NPs	Injection Water (Bacteria) with 100 ppm Biocide
Culture media pre-exposed to endogenous bacteria from the injection water						
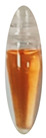	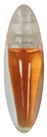	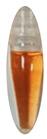	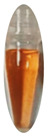	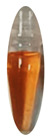	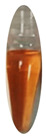	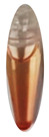
Culture media exposed to endogenous bacteria from the injection water						
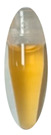	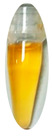	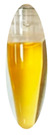	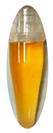	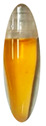	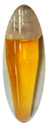	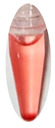
Bacterial viability						
+	+	+	+	+	+	-

## Data Availability

The data presented in this study are available on request from the corresponding author.
